# Validation of a Commercially Available Indirect Elisa Using a Nucleocapside Recombinant Protein for Detection of Schmallenberg Virus Antibodies

**DOI:** 10.1371/journal.pone.0053446

**Published:** 2013-01-15

**Authors:** Emmanuel Bréard, Estelle Lara, Loïc Comtet, Cyril Viarouge, Virginie Doceul, Alexandra Desprat, Damien Vitour, Nathalie Pozzi, Ann Brigitte Cay, Nick De Regge, Philippe Pourquier, Horst Schirrmeier, Bernd Hoffmann, Martin Beer, Corinne Sailleau, Stéphan Zientara

**Affiliations:** 1 Virology Unit, French Agency for Food, Environmental and Occupational Health and Safety, Maisons-Alfort, France; 2 Innovative Veterinary Diagnostic Research and Development, IDVet Society, Montpellier, France; 3 Diagnostic Virology Unit, Friedrich-Loeffler-Institut, Riems, Germany; 4 National Laboratory for the Control of Breeding Animals, Maisons-Alfort, France; 5 Enzootic and (re)emerging Diseases Unit, Veterinary and Agrochemical Research Centre, Brussels, Belgium; USGS National Wildlife Health Center, United States of America

## Abstract

A newly developed Enzym Like Immuno Sorbant Assay (ELISA) based on the recombinant nucleocapsid protein (N) of Schmallenberg virus (SBV) was evaluated and validated for the detection of SBV-specific IgG antibodies in ruminant sera by three European Reference Laboratories. Validation data sets derived from sheep, goat and bovine sera collected in France and Germany (n = 1515) in 2011 and 2012 were categorized according to the results of a virus neutralization test (VNT) or an indirect immuno-flurorescence assay (IFA). The specificity was evaluated with 1364 sera from sheep, goat and bovine collected in France and Belgium before 2009. Overall agreement between VNT and ELISA was 98.9% and 98.3% between VNT and IFA, indicating a very good concordance between the different techniques. Although cross-reactions with other Orthobunyavirus from the Simbu serogroup viruses might occur, it is a highly sensitive, specific and robust ELISA-test validated to detect anti-SBV antibodies. This test can be applied for SBV sero-diagnostics and disease-surveillance studies in ruminant species in Europe.

## Introduction

In 2011, an unidentified disease in cattle was first reported in Germany and the Netherlands. Clinical signs included fever, decreased milk production, and diarrhea. Metagenomic analysis identified a novel Orthobunyavirus, which subsequently was isolated from blood of affected animals [1]. Because of the origin of the first positive samples, the virus was named Schmallenberg virus (SBV). Few months after this first SBV infection, newborns with severe neurological disorders leading mostly to the death of the animal several hours or days after birth and foetuses with atypical malformations leading mostly to intra-uterine death or death immediately after birth were observed. In Europe, 5,234 farms have reported such abnormal newborns or foetuses in cattle (2,865), sheep (2,491) and goats (78) (source: www.survepi.com). Since the detection of this virus, 14 European countries were reported as infected (Belgium, Netherlands, Germany, France, Luxembourg, UK, Denmark, Spain, Italy, Switzerland, Sweden, Austria, Poland and Finland).

Within the family Bunyaviridae, members of the Orthobunyavirus genus involved in the animal health are widely distributed in Asia, Africa, and Oceania but were never observed in Europe. The transmission occurs predominantly through biting midges (Culicoides spp.) and mosquitoes. Viruses in the Simbu serogroup, which includes Akabane, Aino, Shamonda, Sathuperi and now Schmallenberg viruses, have been reported as pathogens of ruminants [2]. Recent phylogenetic analyess clearly show that SBV is closely related to the Sathuperi virus species [1], [3].

The genome of the orthobunyaviruses consists of three RNA (RiboNucleotic Acid) segments. They are designated as small (S) (encoding the nucleocapsid protein (N) and a non-structural protein (NSs) in an overlapping frame), medium (M) (encoding a precursor protein which is cleaved co-translationaly to yield the two surface glycoproteins (Gn and Gc) and a non-structural protein (NSm)) and large (L) (encoding the RNA dependent RNA polymerase) [4]. The Gc protein is an envelope viral protein that induces a specific neutralizing antibody response in infected animals [5]–[8]. Like arenaviruses, the nucleoprotein of bunyaviruses is the most abundant viral antigen present in the virion and in the infected cells [9], [10]. Several serological tests are then often used to detect antibodies against viral nucleoprotein [11]–[15].

Laboratory testing for SBV was necessary to confirm clinical case of these malformations and initially limited to virus or genome detection in malformed offspring. Isolation of infectious virus is currently performed by inoculation of cell lines (e.g. Vero or BHK (Baby Hamster Kidney) 21 cells) with homogenized infected tissues from foetuses or neonates. However, this method is time consuming (3 to 4 days or more). SBV genome can be detected by a real-time Reverse Transcription Polymerisation Chain Reaction (rt-RT-PCR), developed by the FriedrichLoeffler-Institut, and used in all the SBV infected European countries to test biological samples (i.e. brain (for malformed offspring) or blood (during SBV infection in adult ruminant)) [16]. This test is fast, sensitive and specific. However, tissue homogenization, extraction of the viral RNA and amplification by rt-RT-PCR are also time consuming and expensive. While virus isolation and RT-PCR are the most suitable tests to confirm SBV clinical cases, these techniques lead to a serious underestimation of the number of infections. Indeed, to determine the true occurrence and extend of the SBV infection, serological tests are needed. The specific detection of SBV antibodies can be performed by VNT [17] and by immunofluorescence (IF) assays. These methods are time-consuming (in particular VNT, 5 days) and cannot be automated. In order to determine the SBV sero-prevalence in infected countries but also to easily know the serological status of individual animals, it is advantageous to have an easy-to-use assay such as an ELISA method allowing fast screening of large populations. The re-emergence of the disease in the South of France in May 2012 and the fact that SBV is an arbovirus [18] have raised concern that the virus might spread further into non-endemic regions. Serological tools like ELISA assays will be very useful in this context to assess SBV spread.

This paper describes the evaluation and validation of an indirect ELISA for the detection of anti-SBV antibodies.

## Materials and Methods

### Expression and Purification of N Recombinant Protein

The complete N encoding sequence of SBV is available in GenBank (HE649914) and was synthesized and inserted into a plasmid under the control of a T7 promoter. The recombinant plasmids were then transformed into E. coli BL21 DE3 pLysS competent cells. Briefly, E. coli containing the plasmid were grown and the protein expression was induced by addition of IPTG (isopropylthio-β-galactosid) (0.5 mM) for 4 hours at 37°C. Pelleted cells were resuspended in denaturing buffer (Tris 20 mM, NaCl 0.5M, Urea 8M, Lysozyme 0.5 mg/ml) with anti-proteases cocktail (Sigma), sonicated, and centrifuged (30 min, +4°C, 11 000 g). The N recombinant protein containing a N-terminal 6 His-tag was purified from the resulting supernatant using immobilized metal affinity chromatography (IMAC) columns (GE Healthcare, Uppsala, Sweden) according to the manufacturer instructions. Antigen was eluted by acidic shock (Tris HCl) buffer (pH 5.5) and subsequently renatured by a series of dialyses against PBS containing decreasing concentrations of urea (8M, 4M, 2M, 1M, 0M). A complementary steric exclusion chromatography purification step on a HiLoad 26/60 Sephadex 75 column (GE Healthcare, Uppsala, Sweden) was performed. The fractions of interest were concentrated by Vivaspin 5 KD (Millipore, France). The antigen produced was quantified using a BCA (bicinchoninic acid) protein assay kit (Pierce) and its purity controlled by SDS-PAGE (Sodium Dodecyl Sulfate - PolyAcrylamide Gel Electrophoresis).

### Indirect ELISA Procedure for the Detection of Anti-N Antibodies

The N purified protein was then used as antigen in a commercial indirect ELISA kit (SBV indirect ELISA, Id-Vet, France) and all serum samples were tested in the German, Belgian and French laboratories following the manufacturer’s instructions. The results were expressed as S/P values (S/P = (OD sample/ODpositive controle)*100). (S/P<60%: negative; S/P>70: positive and S/P between 60 and 70%: doubtful).

A Receiver Operating Characteristic (ROC) curve, defined by true positive (i.e. sera confirmed SBV positive by VNT) rate (or sensitivity) in function of false positive (i.e. sera confirmed SBV negative by VNT) rate (1-specificity) has been built.

Intra-plate repeatability was evaluated by measuring the coefficient of variation (CV%) of the S/P ratio of 96 repeats of two sera. The measured CV% must be less than 10%. Reproducibility was evaluated by performing the intra-plate repeatability assay on two separate runs. The robustness was also evaluated when the ELISA assays were performed by different operators in different external laboratories.

### Serum Samples

A total of 1364 sera collected in Belgium and in the Brittany, Aveyron and Hérault French regions before 2009 (i.e. before the emergence of SBV in Europe) were used in this study to assess the specificity of the ELISA. These sera were sampled from cattle (n = 780), sheep (n = 496) and goats (n = 88). The Belgian cattle and sheep sera were respectively collected during a leucose screening program in 1997 and a Maedi/Visna screening program in 2005. All sera were stored the entire time at −80°C. The French sera have been collected by departmental laboratories during annual prophylaxies in 2009 and 2010 and stored at −20°C.

Furthermore, a total of 919 sera (Sheep, bovine and goats) collected in 2012 from SBV infected French herds were used to assess the concordance between the ELISA and VNT test and to determine the cut-off value of the ELISA.

In order to assess the concordance between ELISA and IFA, 596 serum samples (395 bovine, 136 ovine, 63 caprine, 3 miscellaneous) collected in Germany within a SBV monitoring program in spring 2012 were used for a comparative investigation in both tests.

### VNT Tests

VNT assays have been performed according to a protocol applied to other seroneutralizing antibodies titrations [19]. Briefly, two-fold dilutions of sera (in duplicates) were prepared in Minimum Essential Medium (MEM, GIBCO), starting at 1∶2 (range of dilution: 2–256). Fifty µl of MEM, containing 100 TCID50 (tissue culture infective dose 50%) of SBV (adapted to Vero cell) were incubated in microtitre plates, with 50 µl of the diluted sera for 1 hour. Then 20 000 Vero cells per well were added in 100 µl of MEM containing 10% fetal calf serum and 2% sodium pyruvate. Microtitre plates were then incubated for 5–6 days at 37°C. Supernatants were removed and 100 µl of absolute ethanol/well were added for cells fixation for 30 minutes. Ethanol was removed and 100 µl of blue methylene (0.1% in water) were added for 30 minutes. Wells with monolayer are blue and wells without are not colored. The neutralizing titer of each serum was defined as the highest dilution allowing neutralization of the 100 TCID50. Titer ≥1/8 (≥0.9 (log10)) was considered as a positive result.

### Immunofluorescence Assay

The IFA was performed in a 96 well multi-plate formate using SBV-infected BHK-21 cells, clone BRS5 (L194, CCLV Insel Riems) as antigen matrix. Briefly, the BHK-21 BSR5 suspension was seeded at 100µl/well and incubated at 37°C in a 2.5% CO2 athmosphere. On the following day (∼ 90% confluence) the odd-numbered columns were inoculated with 100 to 200 TCID50 of SBV each, and the even-numbered columns remained uninfected. After an incubation period of 2 days at 37°C the medium was removed and the plates were fixed using heat treatment (2 h at 80°C). Both one infected and one control well were incubated with each serum sample in a 1∶100 dilution for 1 hour at room temperature. After a washing step with TBST, a second incubation period with an FITC (Fluoresceine IsoThioCyanate) -labelled anti-species conjugate (Anti Bovine IgG-FITC or Anti-sheep IgG-FITC, Sigma, Germany) was performed. Subsequently, the monolayer was washed repeatedly, embedded with Dabco fluorescence conservation buffer, and analysed with an inverted fluorescence microscope (IX50 Olympus).

## Results

### Expression and Purification of SBV Nucleoprotein

**Figure 1:**
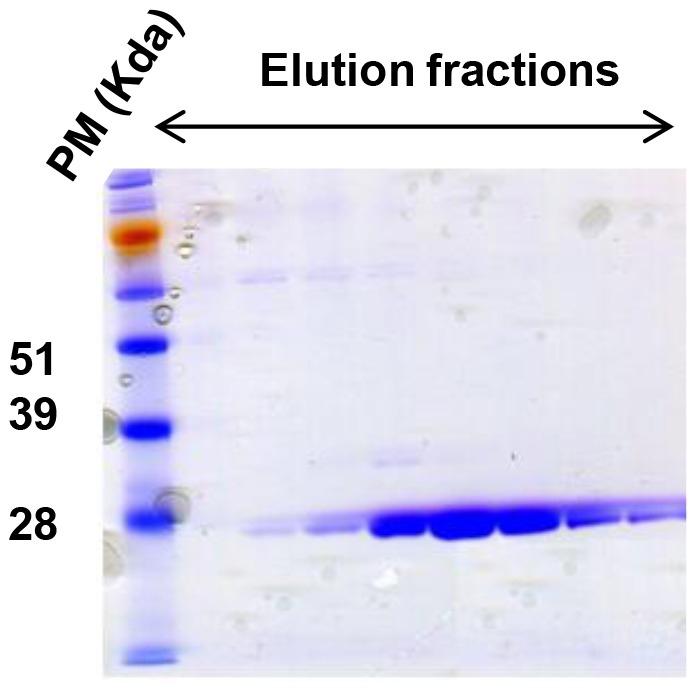
Coomassie blue staining of the N protein. After purification steps, different elution fractions of purified N recombinant protein were separated by SDS-PAGE in a 12% polyacrylamide gel and stained with coomassie blue.

After the purification of the recombinant N protein by steric exclusion chromatography, the purity of the production was controlled by SDS-PAGE (Figure 1). Results showed that fractions 3, 4, 5 and 9 had the most protein concentrations. As for purity, fractions 4 and 5 showed no detectable background contaminants.

### ELISA Validation

**Figure 2:**
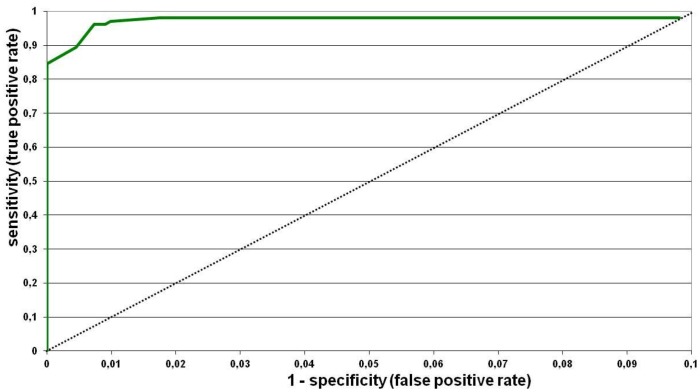
ROC curve. The curve presents the true positive rate (or sensitivity) in function of false positive rate for different cut-off points. Each point on the ROC curve represents a sensitivity/specificity pair corresponding to a particular decision threshold.

**Figure 3:**
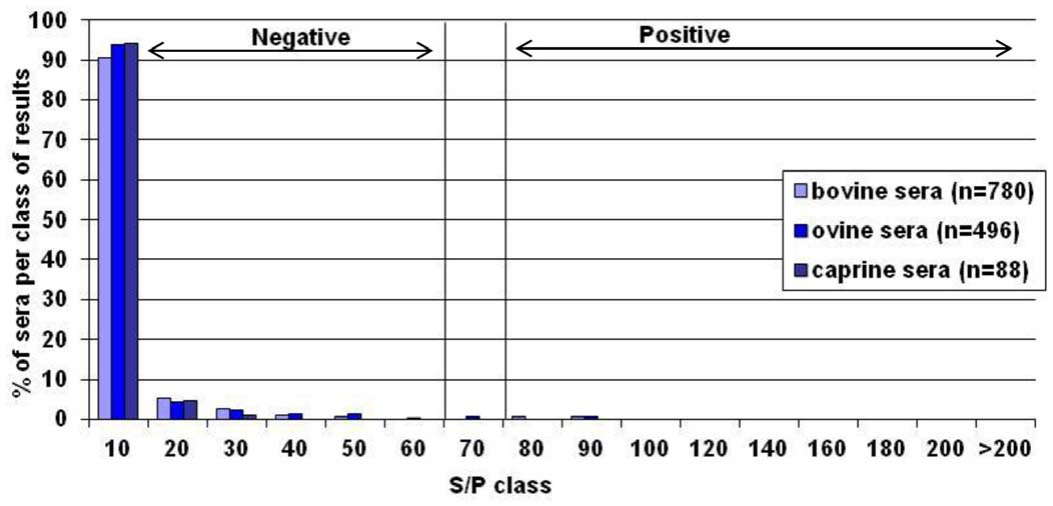
Distribution of the percentage of sera per class of results. The S/P distribution for 1364 SBV negative sera was represented in function of S/P results.

To determine the cut-off value of the ELISA, the S/P values of the 1364 sera collected before 2009 (considered SBV negative, all were VNT negative) and 180 VNT positive sera from infected French herds (bovine, ovine or goats) were used to built the ROC curve (Figure 2). The cut-off was determined at an S/P value of 60%, corresponding to a specificity of 99.8% and sensitivity of 97.2%. The respective S/P distribution for these negative sera is given in Figure 3.

### ELISA and VNT Comparison

Sera having a VNT titer equal or greater than 0.9 log10 (dilution 1∶8) were considered as positive by ANSES. A total of 919 sera from infected French herds were tested by VNT (ANSES, Maisons-Alfort, France and LNCR/ACSEDIATE, Maisons-Alfort, France).

Out of 919 sera tested, 909 sera had the same results on both techniques, giving an agreement of 98.9%, demonstrating an excellent concordance between the ELISA assay and the VNT. The results are presented in the contingency table (Table 1).

**Table 1:** Concordance between the ELISA S/P value and VNT titer.

		SNT		
		positive	negative	TOTAL
**ELISA**	**positive**	164	2	**166**
	**Doubfull**	2	1	**3**
	**negative**	5	745	**750**
	**TOTAL**	**171**	**748**	**919**

The Table presents the results of 919 sera analyzed by ELISA and VNT.

### ELISA and IFA Comparison

In Germany, 596 sera were collected and characterized by the SBV-specific IFA. One hundred and forty six sera were identified as positive and 450 were classified as negative (Table 2). Out of those 596 sera, the status of 561 sera obtained by both techniques (ELISA and IFA) was identical, resulting in a concordance between both tests of 94.1%. Twenty five sera scored positive in the ELISA and negative in the IFA, while 10 samples were ELISA-negative and IFA positive.

**Table 2:** Concordance between the ELISA S/P value and IFA results.

		IFA		
		positive	negative	TOTAL
**ELISA**	**positive**	136	25	**161**
	**negative**	10	425	**435**
	**TOTAL**	**146**	**450**	**596**

The Table presents the results of 596 sera analyzed by ELISA and IFA.

### Repeatability, Reproducibility and Robustness of the SBV ELISA

Intra-plate repeatability was evaluated by measuring the CV% of 96 repeats of the ELISA positive control and a weak positive serum. The CV obtained was 7% and 9% respectively (Data not shown). For the reproducibility, CV was 8% for the positive control serum and 10% for the weak serum (Data not shown).

The robustness (or inter-laboratory reproducibility) of the kit was also evaluated: the S/P values of a weak positive serum tested in 10 independent tests showed that they were always in the range of 50–70%, demonstrating a good robustness of the ELISA (Data not shown).

## Discussion

In many European countries, the notification of suspected SBV cases (occurrence of arthrogryposis hydranencephaly syndrome in calves, lambs, and goat kids) is compulsory. A clinical report must then be followed by a laboratory test for confirmation. During the first months following the discovery of this new virus, case confirmation was done by the detection of viral RNA using rt-RT-PCR in brain tissue sampled from death newborns. However, because viral RNA can be destroyed (e.g. depending on the time of the sampling in the dead animal or depending on the transport and storage conditions) the clinical case number was underestimated. In addition, infected livestock may give birth to healthy young animals, adding to the underestimation of the true rate of infection. All together, these results obtained with virus or genome detection underestimated the number of infections and therefore, sero-diagnostic studies are needed to determine the real exposure of the ruminant population to SBV in affected countries. The first sero-prevalence studies detected the presence of antibodies against SBV using a virus neutralization test [20]. More recently, an evaluation of a VNT protocol (very similar to our VNT protocol described in this paper) demonstrated that this test can be used for testing of animals for export, surveillance, screening and research purposes, but can also be used as a confirmation test for commercially available enzyme-linked immunosorbent assays (ELISA’s) [17]. Nevertheless, 5 days are required to obtain results using this test and a high throughput is not feasible. It is therefore of great interest to have a rapid and specific serological assay such as an ELISA available for high throughput testing e.g. in monitoring and surveillance studies but also for the export/import testing of ruminants.

Using the same strategy developed for Rift valley fever ELISA, the N viral protein of SBV has been expressed in a prokaryote system with a 6 histidine tag [14]. After expression, denaturation in the presence of urea, purification by IMAC chromatography and some desalting steps, the SBV N recombinant protein was used as antigen to discriminate sera from SBV infected from naive animals. Purification steps are particularly important to decrease the non-specific signals, due to E. coli proteins that are still present after purification. In this case, the non specific results are lower than 0.25%, suggesting a single doubtful or a false positive result for 400 samples. The ROC curve illustrates well the high specificity and sensibility of this first SBV specific ELISA test commercially available.

Some cross reactions between nucleocapsid protein and IgG antibodies induced by infection with other Simbu serogroup virus members might occur [14]. Limited cross reactions were observed with sera from ruminants experimentally infected with Akabane virus (AKAV) (data not shown). However, sera sampled from different cattle, sheep or goat livestock in France or Belgium before 2011 that were used to evaluate the ELISA specificity showed no cross reaction as expected as no Simbu serogroup viruses were present in this area before the SBV incursion in 2011 [20]. Since June 2012, the spread of SBV has been also confirmed in cattle in the South of France. Viraemic blood samples with high levels of SBV RNA genome were detected by rt-RT-PCR. Serological studies of these newly infected cases demonstrated that specific IgG against SBV N could be detected by the presented ELISA ten days after genome detection in the blood (Data not shown).

The sensitivity of the ELISA was evaluated with sera received for SBV diagnosis. Their infection status was determined using VNT or IFA. The agreement between VNT and ELISA was 98.9%, indicating a very good concordance between the two serological assays. A similar study in Germany displayed corresponding results between VNT and ELISA (98.5% of strong positive sera; personal data). However, about 50% of samples with an S/P value between 60 and 70% (classified as questionable) displayed a low titer (1∶5 to 1∶20) in the VNT. For the evaluation it has to be also considered that the ELISA test and the VNT detect antibodies against different antigenic determinants. While the ELISA detects antibodies against the N protein of SBV, the VNT measures the neutralizing antibodies induced by the viral glycoproteins Gn and Gc. Therefore, deviating results in a small range could be also explained by those test specific differences.

It has to be also mentioned that 25 IFA negative sera were characterized as positive by ELISA. With regard to the specificity of the test, these sera were finally assumed as true positives (based on epidemiological data and VNT results), demonstrating a higher sensitivity of the ELISA test in comparison to the used IFA.

The different assays performed in different laboratories in Germany and France have shown a high intra- and inter-plate repeatability when comparing the values of the S/P obtained with the positive and negative sera during the validation procedures. All these data demonstrate the robustness of the indirect ELISA protocol, showing that this first SBV specific ELISA is a fast and reliable test, also for large scale SBV serological studies.
